# Quality assurance and long‐term stability of a novel 3‐in‐1 X‐ray system for brachytherapy

**DOI:** 10.1002/acm2.13727

**Published:** 2022-07-18

**Authors:** Andre Karius, Juliane Szkitsak, Vasilios Boronikolas, Rainer Fietkau, Christoph Bert

**Affiliations:** ^1^ Department of Radiation Oncology Universitätsklinikum Erlangen, Friedrich‐Alexander‐Universität Erlangen‐Nürnberg Erlangen Germany; ^2^ Comprehensive Cancer Center Erlangen‐EMN (CCC ER‐EMN) Erlangen Germany; ^3^ Abteilung für medizinische Physik, Klinik für Strahlenheilkunde Universitätsklinikum Freiburg Freiburg im Breisgau Deutschland; ^4^ Medizinische Fakultät Albert‐Ludwigs‐Universität Freiburg Freiburg im Breisgau Deutschland; ^5^ Partnerstandort Freiburg Deutsches Konsortium für Translationale Krebsforschung (DKTK) Freiburg im Breisgau Deutschland; ^6^ Partnerstandort Freiburg Deutsches Krebsforschungszentrum (DKFZ) Heidelberg Deutschland

**Keywords:** 3‐in‐1 X‐ray system, cone‐beam computed tomography, planar imaging, quality assurance

## Abstract

**Purpose:**

A novel, mobile 3‐in‐1 X‐ray system featuring radiography, fluoroscopy, and cone‐beam computed tomography (CBCT) has been launched for brachytherapy recently. Currently, there is no quality assurance (QA) procedure explicitly applicable to this system equipped with innovative technologies such as dynamic jaws and motorized lasers. We developed a dedicated QA procedure and, based on its performance for a duration of 6 months, provide an assessment of the device's stability over time.

**Methods:**

With the developed QA procedure, we assessed the system's planar and CBCT‐imaging performance by investigating geometric accuracy, CT‐number stability, contrast‐noise‐ratio, uniformity, spatial resolution, low‐contrast detectability, dynamic range, and X‐ray exposure using dedicated phantoms. Furthermore, we evaluated geometric stability by using the flexmap‐approach and investigated the device's laser‐ and jaw‐positioning accuracy with an in‐house test phantom. CBCT‐ and planar‐imaging protocols for pelvis, breast, and abdomen imaging were examined.

**Results:**

Planar‐ and CBCT‐imaging performances were widely stable with a geometric accuracy ≤1 mm, CT‐number stability of up to 46 HU, and uniformity variations of up to 48 HU over time. For planar imaging, low‐contrast detectability and dynamic range exceeded current recommendations. Although geometric stability was considered tolerable, partly substantial positioning inaccuracies of up to more than 120 mm and −13 mm were obtained for lasers and jaws, respectively. X‐ray exposure showed small variations of ≤0.56 μGy and ≤0.76 mGy for planar‐ and CBCT‐imaging, respectively. The conductance of the QA procedure allowed a smooth evaluation of the system's overall performance.

**Conclusion:**

We developed a QA workflow for a novel 3‐in‐1 X‐ray system allowing to assess the device's imaging and hardware performance. The system showed in general a reasonable imaging performance and stability over time, whereas improvements regarding laser and jaw accuracy are strictly required.

## 1 INTRODUCTION

In recent years, several digital X‐ray systems featuring radiography, fluoroscopy, and cone‐beam computed tomography (CBCT) integrated into one device have been launched.^1^, ^2^, ^3^, ^4^ These 3‐in‐1 systems are principally capable of covering a wide range of imaging tasks. For instance, broad applications have already been reported for head–neck surgery,^1^ maxillofacial surgery,^5^, ^6^ dentistry,^7^, ^8^ and radiotherapy.^9^, ^10^, ^11^, ^12^ To ensure performance stability in clinical operation, conducting technical quality assurance (QA) is essential.

To date, a number of QA guidelines exist for planar‐ and CBCT‐imaging.^13^, ^14^, ^15^, ^16^ Despite, there is currently no gold‐standard for the QA of mobile 3‐in‐1 X‐ray systems. The mentioned guidelines also mainly focus on image quality considerations, but in general do not address hardware features of examined devices. Particularly innovative technologies, such as dynamic jaws and motorized lasers require, in our opinion, explicit assessments in dedicated QA procedures.

In the present work, we developed a QA workflow for a novel 3‐in‐1 X‐ray system (ImagingRing m, IRm; medPhoton, Austria) for brachytherapy. Our workflow includes both assessments of its radiography, fluoroscopy, and CBCT‐imaging performance regarding image quality and dose as well as evaluations of its geometric stability, dynamic jaws, and movable lasers. As the procedure was carried out weekly for 6 months, we also provide an assessment regarding the IRm's stability over time.

## 2 METHODS

### 2.1 ImagingRing m (IRm)

**FIGURE 1:**
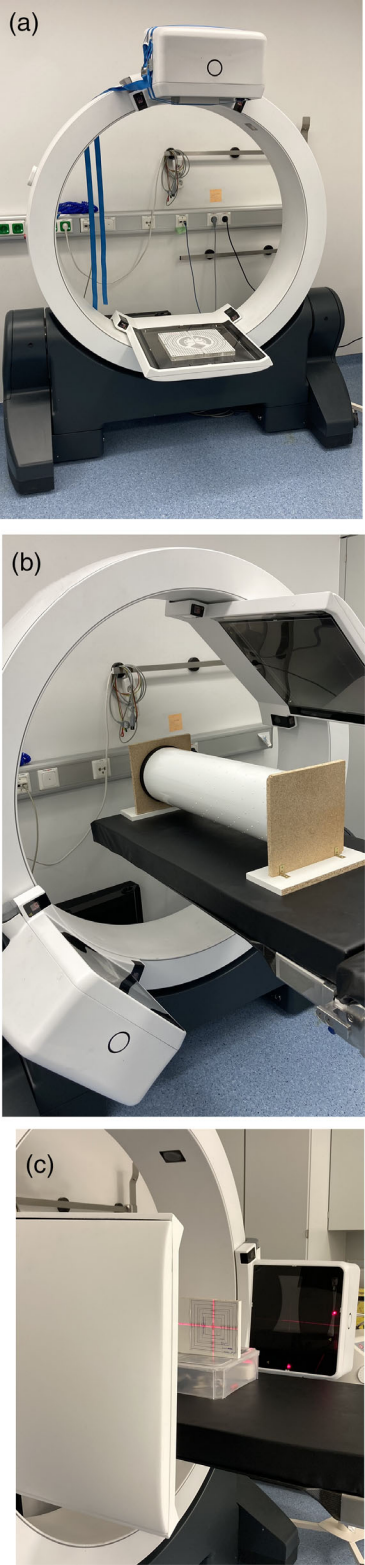
ImagingRing m (IRm) and (a) the phantom setup used to evaluate planar imaging performance (NORMI RAD/FLU placed on the detector, with 16.3 × 16.3 cm^2^ aluminum block of 2.5 cm thickness being mounted in front of the source exit window), (b) the flexmap phantom fixed to a stable holder to prevent rolling used to analyze geometric stability, and (c) the in‐house test phantom for assessing laser and jaw accuracy

A detailed technical description of the IRm (Figure 1a–c) was provided by Karius et al.^9^, ^12^ The device features particularly 121‐cm gantry clearance, a 43.2 × 43.2‐cm^2^ flat‐panel detector (FPD), and the capability for non‐isocentric imaging by independently movable X‐ray source and FPD. Source and detector can be rotated independently from each other by more than 650° along the gantry in both clockwise and counter‐clockwise directions, and arranged at any position with 0.1° accuracy. Planar‐ and CBCT‐imaging is feasible with a tube voltage of 60–120 kV (in 10 kV steps) with continuous and pulsed (pulse length: 2–35 ms, tube‐current: 5–120 mA) tube output. The device is handled via Wi‐Fi remote control by means of a portable control unit. All device movements (longitudinal/lateral translations, rotations, tilt up to ±30°) can be performed battery‐powered for up to 30 min. Patient monitoring is enabled by four integrated optical cameras.

Furthermore, the IRm features four independently, dynamically collimating jaws. These allow to limit the field of view (FOV) of examinations to the anatomical region of interest (ROI). In a so‐called volume‐definition workflow,^9^, ^12^ anterior–posterior (AP) and lateral (LAT) topograms are acquired first and allow for a subsequent FOV definition of the actual CBCT‐scan. Six different prefilters (air; 1.5 mm Al; 0.2, 0.5, and 1.5 mm Cu; Cu‐BowTie) allow beam quality adjustments. Four line‐lasers, which can move motorized on rails along the FPD edges, enable FOV visualizations on the patient's skin. The lasers can display both a crosshair and rectangular target. In the latter case, the jaw positions are virtually projected into the isocentric plane, and, hence, the target‐laser edges indicate the outer limits of acquired X‐ray images.

### 2.2 QA workflow: overview

For the QA procedure, we focused on protocols for breast, pelvis, and abdomen imaging. Scan parameters are summarized in Table 1.

**TABLE 1:** List of the main parameters of the scan protocols used for planar and cone‐beam computed tomography (CBCT)imaging

CBCT protocol	Scan time (s)	Tube voltage (kV)	Total tube output (mAs)	Frames	Pre‐filter	Kernel (cutoff)
Pelvis	25	120	198	300	0.2 mm Cu	Cosine (0.9)
Breast	17	110	171	204	0.5 mm Cu	Shepp–Logan (0.8)
Abdomen	50	120	240	600	Cu BowTie	Hamming (0.7)

Planar protocols	Detector binning	Tube voltage (kV)	Total tube output (mAs)	Frames	Pre‐filter	
Pelvis	3 × 3	100	3.5	5	1.5 mm Cu	
Breast	2 × 2	80	1.1	11	1.5 mm Al	
Abdomen	2 × 2	90	2.5	3	0.5 mm Cu	
Fluoroscopy	2 × 2	110	112	179	0.5 mm Cu	

*Note*: For CBCT‐imaging, the reconstruction kernels are specified with the selected ramp filter cutoff (unit: fraction of the Nyquist frequency).

For breast and pelvis CBCT‐imaging, short scans (180°+beam divergence) with source right orbit were used. This means that—if a patient lies with his/her head positioned on the source/detector‐opposing side of the gantry on the couch—the X‐ray tube rotated by 180° along the right‐hand side of the patient, with the source rotation starting above the patient. The pelvis protocol exhibited 2.5:1 velocity modulation, thus reducing the travel speed by a factor of 2.5 in the lateral regime. The abdomen protocol referred to a stitched full scan (360°), again with 2.5:1 velocity modulation. This means that two laterally shifted FOVs are imaged within one single CBCT‐acquisition, which are stitched together during reconstruction. This allows FOV extensions in lateral direction.^17^ Scans were acquired with 12‐Hz frame rate, pulsed tube output, 2 × 2 detector binning (pixel pitch: 300 µm), and 0.4 × 0.4 × 1 mm^3^ voxel size, unless otherwise mentioned.

The QA procedure was carried out weekly for 6 months. Exceptions were 2 weeks after the fourth measurement (vacation) and 3 weeks after the fifth measurement (maintenance by manufacturer). The manufacturer's maintenance resulted in improved scatter handling for stitched full scans, as well as post‐processing adjustments in planar imaging with additional contrast‐enhancing/noise reduction being applied to the raw images. The variations of each evaluated parameter were assessed over time.

### 2.3 CBCT‐imaging

**FIGURE 2:**
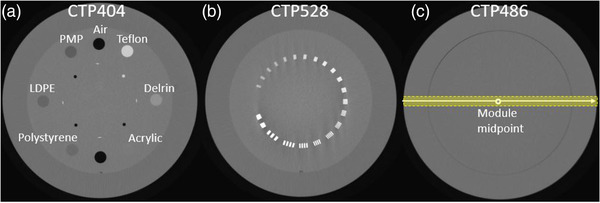
CatPhan phantom: Part (a) provides the CTP404 module with each insert being labeled, (b) the 21 line pair of the CTP528 module, and (c) the CTP486 homogeneity module used for uniformity analysis. For the latter, 11 image rows (marked yellow) were averaged and afterwards a CT‐number profile along the lateral direction was drawn. Window level: 150 HU. Width: 2500 HU

For the CBCT‐QA, the CatPhan 504 (Figure 2a–c; CatPhan; The Phantom Laboratory, USA) was used. The phantom was placed isocentrically within the IRm and scanned with each CBCT‐protocol. Image quality was assessed as follows.

#### 2.3.1 Geometric accuracy

The four rods on the central slice of the module CTP404 (Figure 2a) were detected via thresholding by using the software QAMaster.^18^ The software determines the side and diagonal lengths of the rod quadrilateral as Euclidean distances between the corresponding centers and compares them to manufacturer's specifications.

#### 2.3.2 CT‐number stability

A circular ROI was positioned within each CTP404 insert, and the respective mean CT numbers $CT_{insert}$ were measured. The ROIs had a radius 1 mm smaller than the insert radius to account for CT‐number fluctuations at the insert edges^18^.

#### 2.3.3 Contrast–noise ratio (CNR)

Additional ROIs were placed adjacent to the three inserts with the lowest subject contrast (LDPE, polystyrene, and acrylic) in the CTP404 background. Mean $CT_{bkg}$ and standard deviation $\sigma_{bkg}$ of the corresponding CT numbers were determined and served for contrast‐noise‐ratio (CNR) calculations:

(1) $$CNR_{insert}=\frac{\left|CT_{insert}-CT_{bkg}\right|}{\sigma_{bkg}}.$$

#### 2.3.4 Spatial resolution

For each of the 21 line‐pairs (lp) (frequency: 1–21 lp/cm) of the CTP528 module (Figure 2b), the modulation was calculated according to the Droege–Morin method^19^, ^20^ to obtain the modulation transfer function (MTF).

#### 2.3.5 Uniformity

As illustrated in Figure 2c, the 11 image rows centered around the horizontal midline through the CTP486 module's central slice were averaged, and afterward a CT‐number profile along the lateral direction was drawn. Uniformity was calculated as difference between the CT numbers of profile edge (at 95% of the profile length) and midpoint.

#### 2.3.6 Weighted cone‐beam dose index (CBDI_w_)

To evaluate dose stability, an IEC 60601‐2‐44 compliant body dosimetry phantom^21^ was placed isocentrically within the scanner on a carbon fiber table. A 10 cm pencil chamber (type 30009; PTW‐Freiburg, Germany) was sequentially inserted into the four peripheral and the central phantom boreholes, and the corresponding dose length integrals were measured. Based on this, the weighted cone‐beam dose index (CBDI_w_) was calculated according to Buckley et al.^22^ Weekly CBDI_w_ measurements were performed for the pelvis protocol only. This was done to save time requirements in clinical routine and in order to not exceed the tolerable heat‐load of the IRm by performing multiple CBDI_w_ measurements for each protocol.

### 2.4 Planar‐imaging

For the planar‐imaging QA, the NORMI RAD/FLU (300 × 300)mm T42032 (Figure 1a, PTW‐Freiburg, Germany) was used. A 16.3 × 16.3 cm^2^ aluminum block with 2.5 cm thickness supplied with the phantom was mounted in front of the X‐ray source exit window for patient simulation, and the phantom was placed centered on the detector. It was imaged once with each planar‐imaging protocol. The following parameters were evaluated:

#### 2.4.1 Spatial resolution

For MTF determination, the phantom's 20 line‐grids (frequency *f*: 0.6–5.0 lp/mm) were considered. For each grid, modulation *M* was calculated as

(2) $$M\;\left(f\right)=\frac{P_{95}\left(f\right)-P_{5}\left(f\right)}{P_{95}\left(f\right)+P_{5}\left(f\right)}.$$

$\mathrm{where}\;P_{95}\;$ and *P*_5_ refer to the 95th and 5th pixel value percentile of each grid, respectively. Measurements were normalized to the “real” grid contrast resulting from the maximum pixel value of the first grid and the mean pixel value of a background ROI.

#### 2.4.2 Dynamic range

A ROI was positioned within each of the 17 steps of the phantom's dynamic staircase, and the corresponding pixel mean values and standard deviations were measured. If the mean value of a step differed more than three standard deviations from the means of its neighboring steps, it was rated detectable. Dynamic range was determined as the total number of steps being detectable. Note that not all steps may become visible on the scans. Therefore, a mask with their known positions was put over the acquired images for the measurements.

#### 2.4.3 Low‐contrast detectability

A circular ROI with 1.5 mm radius was placed within and adjacent to each of the phantom's eight detail objects. The corresponding pixel mean values and standard deviations were measured, and the CNR was calculated according to Equation (1). Note that not all objects may become visible on the scans. Therefore, a mask with their known positions was put over the acquired images for the measurements.

#### 2.4.4 Detector entrance dose

Detector entrance dose was determined by placing a 3.2 cm^3^ solid state detector (QUART dido 2000K; QUART, Germany) centered onto the NORMI RAD/FLU phantom. X‐ray exposure was measured for each protocol.

### 2.5 Geometric stability

Geometric calibration of the IRm was performed using the flexmap‐approach by means of a nine‐degree‐of‐freedom ball bearing phantom supplied with the IRm, which was placed isocentrically within the scanner (Figure 1b). During the calibration procedure that has been described in detail by Karius et al.^12^ and Keuschnigg et al.,^23^ three translational corrections of source (tS*_x_*_,_ tS*_y_*, tS*_z_*) and detector (tC*_x_*_,_ tC*_y_*, tC*_z_*) as well as three detector angular corrections (r*_x_*, r*_y_*, r*_z_*) are determined. *x* denotes the gantry's rotation direction, *y* the direction along the rotational axis, and *z* the radial direction within the tomographic plane. The procedure is performed separately for clockwise and counter‐clockwise rotation directions. Based on the weekly calibrations, the variations of the obtained corrections were assessed as a measure for geometric stability.

### 2.6 Laser accuracy

For laser validation, an in‐house test phantom (Figure 1c) was used. On the phantom, squares of different sizes as well as the phantom center are marked visually and by X‐ray markers. The phantom center was aligned to the crosshair‐laser within the isocentric plane, and a 10 × 10 cm^2^ X‐ray image was acquired. The resulting Euclidean distance between image center and phantom center provided a measure for the crosshair‐laser's accuracy.

Afterward, the target‐laser was set to project field sizes of 10 × 10 cm^2^, 12 × 12 cm^2^, and 14 × 14 cm^2^ into the isocentric plane. The set field sizes were compared to the ones that were actually projected onto the phantom.

All laser tests were performed for both the IRm's AP (i.e., source at 6 o'clock and detector at 0 o'clock position) and LAT (i.e., source at 3 o'clock and detector at 9 o'clock position) imaging position.

### 2.7 Jaw accuracy

To assess jaw accuracy for planar imaging, the laser test phantom was centered within the isocentric plane, and X‐ray images with field sizes of 10 × 10 cm^2^, 12 × 12 cm^2^, and 14 × 14 cm^2^ were acquired. The comparison between set field sizes and actually measured field sizes allowed a validation of jaw positioning accuracy. These checks were performed for both the AP and LAT imaging position.

For CBCT‐imaging, the CatPhan phantom was positioned isocentrically within the scanner. The jaws were set within the volume‐definition‐workflow in such a way that the outer boundary of the CBCT‐FOV matched the phantom margins. For imaging, the breast protocol was used. For each pixel belonging to the phantom‐boundary, the Euclidean distance to the phantom center was calculated on the acquired scan. The difference between these distances and the actually set FOV‐size (and thus the phantom's radius) yielded a measure for jaw accuracy during CBCT‐imaging.

## 3 RESULTS

The baseline results obtained for each imaging parameter during the first measurement are provided as the Supporting Information A for each scan protocol. The results obtained after maintenance, and thus the reacquired baselines, are also shown in the Supporting Information A to illustrate the impact of the maintenance on the stitched full scans and planar imaging.

### 3.1 CBCT‐imaging

**FIGURE 3:**
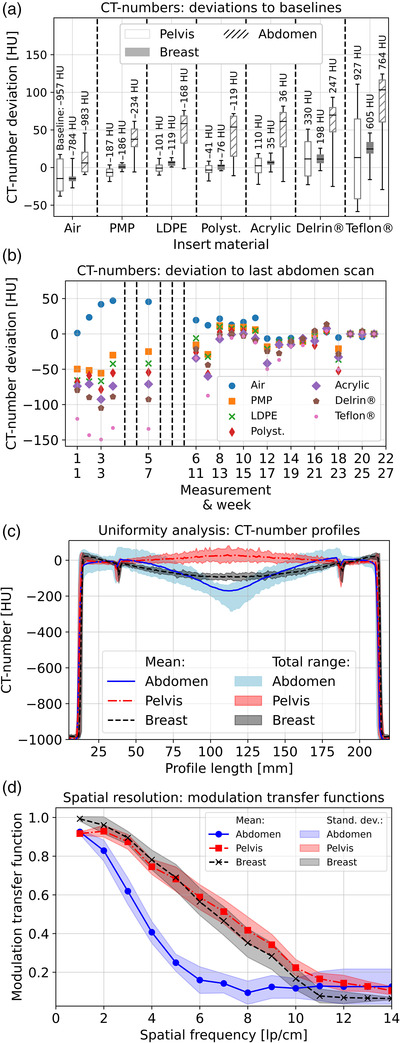
Analysis of cone‐beam computed tomography (CBCT) imaging performance: Part (a) shows for each insert and protocol the CT‐number deviations to the respective baselines (obtained in the very first measurement) considering all measurements. The horizontal lines in the boxes correspond to the median of the results, the boxes represent the interquartile range (25th–75th percentile), and the whiskers show the total range of the results (minimum to maximum value). Part (b) visualizes the corresponding CT‐number deviations to the last measurement for the abdomen protocol. The dashed lines indicate the weeks in which no measurements could be performed (see Section 2.2). In (c) the obtained CT‐number profiles are provided. The lines represent for each protocol the mean profile based on all 21 conducted measurements, in which each single measurement is the mean of 11 line profiles, as described in Section 2.3. Shadowing represents the range of the results over time. Part (d) provides the calculated modulation transfer functions (MTFs), with the shadowing representing the standard deviations.

The CBCT‐imaging performance showed a reasonable stability over time. The CT numbers deviated by a maximum of 51 HU (Delrin) and 46 HU (Teflon) from the baselines for the pelvis and breast protocol, respectively (Figure 3a). An exception for pelvis imaging was Teflon, for which deviations up to 110 HU were measured. CT numbers obtained with the abdomen protocol deviated stronger from the baselines. However, considering the corresponding differences to the last measurement week (Figure 3b), these substantial deviations originated from strongly reduced CT numbers during the first five measurements. In the time after, CT‐number stability was comparable to the pelvis/breast protocols, and the results were in good agreement with manufacturer specifications.^24^ Concomitant with this, the CNR was also widely stable over time. As an example, mean CNRs of 13.2 (11.0–15.0)/9.9 (7.8–12.7)/2.25 (1.0–3.6) were measured for the LDPE/polystyrene/acrylic insert using the pelvis protocol, respectively. A similar CNR stability was also obtained for breast imaging. Abdomen imaging revealed mean CNRs of 12.1 (7.9–18.1)/14.6 (9.8–17.6)/1.9 (1.0–3.1) prior to maintenance and 14.1 (11.1–21.1)/18.0 (13.1–23.4)/2.0 (1.5–3.0) after manufacturer maintenance for the LDPE/polystyrene/acrylic insert, respectively.

Regarding image uniformity, a cupping of 110 HU (range: 88–136 HU) and −35 HU (−72 to −7 HU) widely stable over time was observed for the breast and pelvis protocol, respectively (Figure 3c). The abdomen protocol showed an overall uniformity of 165 HU (124–228 HU) (after week 5: 154 HU, range: 124–167 HU; before week 5: 200 HU, range: 132–228 HU). The increased cupping of the first five measurements explained the substantial CT number deviations of the CTP404 inserts reported earlier.

The calculated MTFs were similar for the breast and pelvis protocol, with the limiting resolution (frequency where MTF drops to 0.1) being slightly increased for the latter (Figure 3d). In comparison, the resolution characteristics for abdomen imaging were substantially reduced with a limiting resolution of only about 7–8 lp/cm, despite significantly extended scan time. Basically, no differences between the time before and after maintenance were found. The corresponding images showed slight overall blurring, and thus the observed reduction was not only attributable to the used smooth kernel. However, basically no differences were found between the various protocols regarding geometric accuracy. The deviations between measured and actual rod distances were ≤1 mm in each case, with the largest deviations measured along the quadrilateral‐diagonal. With a pixel‐diagonal of $\sqrt{2}\;\times \;$0.4 mm = 0.56 mm, these were attributable to partial volume effects.

**FIGURE 4:**
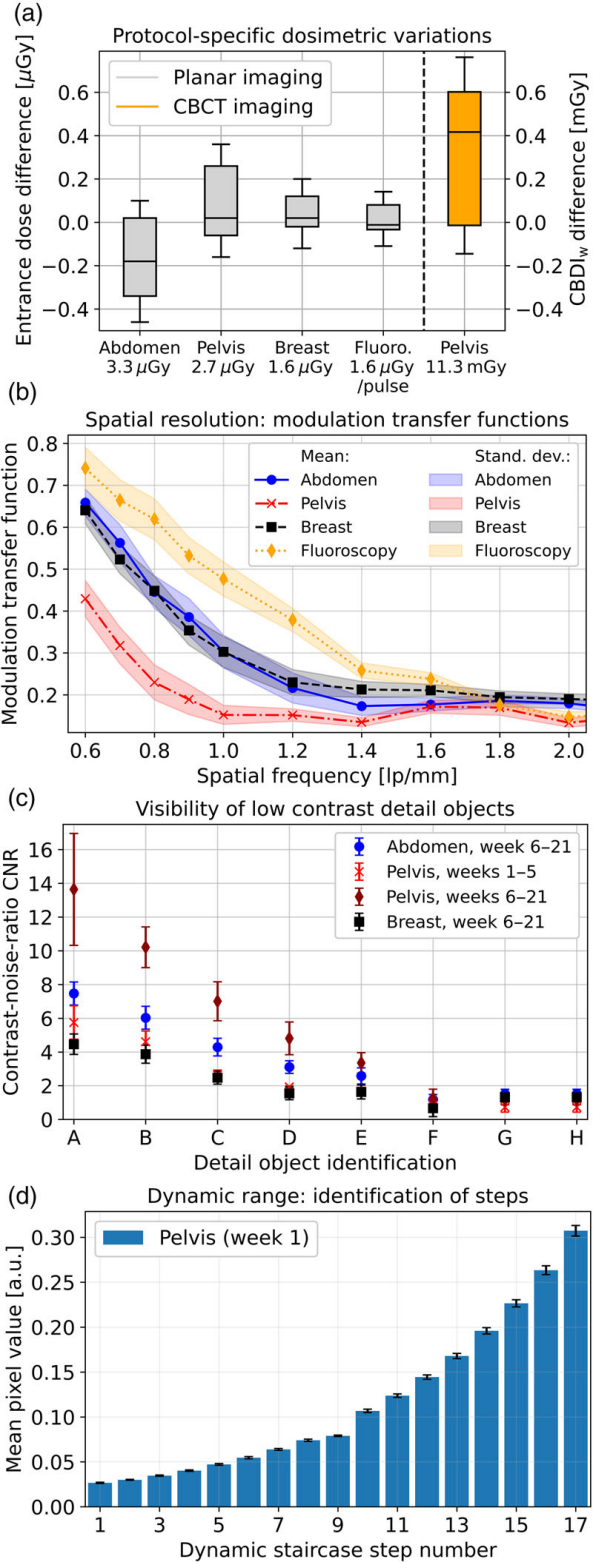
Evaluation of dose stability and planar imaging: Part (a) shows the dosimetric differences to the respective baselines for the planar protocols and the pelvis cone‐beam computed tomography (CBCT) protocol obtained over time. The baselines obtained during the very first measurement is provided below each protocol label. For clarity of illustration, the dosimetric variation for fluoroscopy is provided per pulse. The horizontal lines in the boxes correspond to the median of the results, the boxes represent the interquartile range (25th–75th percentile), and the whiskers show the total range of the results (minimum to maximum value). Part (b) provides the measured modulation transfer functions (MTFs) for each protocol and (c) gives the contrast‐noise‐ratio (CNR) determined for the individual detail objects. Error bars refer to the corresponding standard deviations. Note that the CNR results differed strongly depending on the measurement weeks, as exemplarily shown for the pelvis protocol. Part (d) shows the pixel value mean and standard deviation for each staircase step, exemplarily for the pelvis protocol in week 1.

Regarding the visual impression of scans, weekly varying misalignment and streak artifacts were observed. Corresponding CTP404 images are exemplarily provided as the Supporting Information B. The artifacts seemed to have only minor impact on the quantitative image analysis but impaired the overall image quality partly significantly. Finally, the CBDI_w_ measurements for the pelvis protocol revealed a stable X‐ray exposure with a maximum deviation of 0.76 mGy to the baseline of 11.32 mGy (Figure 4a).

### 3.2 Planar‐imaging

Stable MTFs were also obtained in examining the planar‐imaging performance (Figure 4b). For the abdomen, breast, and fluoroscopy protocol, a limiting resolution (frequency at which MTF starts saturating) of about 1.2 lp/mm, 1.2 lp/mm, and 1.8 lp/mm was obtained, respectively. Due to the larger detector binning, the pelvis protocol revealed a reduced resolution characteristic with a limiting resolution of only 1.0 lp/mm. Maintenance did not reveal any significant effects on resolution characteristics.

In examining low‐contrast detectability and dynamic range, significant improvements were obtained after the fifth measurement week. Although the number of identifiable detail objects did not increase over time (five objects were detectable for each protocol), their CNR became strongly enhanced by substantial reductions of image noise. This is illustrated in Figure 4c, which shows a CNR increase of factor 2–3 exemplarily for the pelvis protocol. A comparable behavior was also observed for all other protocols examined. For assessing dynamic range, the pixel mean values and standard deviations were measured for each staircase step (Figure 4d). Prior to maintenance, the baselines of 11, 16, 15, and 14 steps were detected for the breast, pelvis, abdomen, and fluoroscopy protocol, respectively. After the fifth week_,_ all 17 steps were detectable for each protocol.

Detector entrance dose was determined to a mean of 3.1 μGy (range: 2.8–3.36 μGy), 2.8 μGy (2.58–3.1 μGy), 1.63 μGy (1.46–1.78 μGy), and 1.57 μGy/pulse (1.45–1.70 μGy/pulse) for the abdomen, pelvis, breast, and fluoroscopy protocol, respectively, and thus proved to be stable over time (Figure 4a).

### 3.3 Geometric stability

**FIGURE 5:**
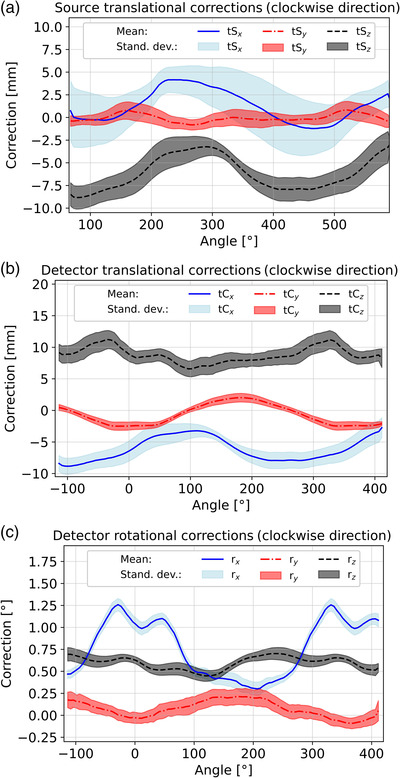
Evaluation of geometric stability for the clockwise gantry rotation direction: Part (a) shows the three translational corrections of source (tS*_x_*, tS*_y_*, tS*_z_*) and (b) of the detector (tC*_x_*, tC*_y_*, tC*_z_*). Part (c) provides the three detector angular corrections (r*_x_*, r*_y_*, r*_z_*). The shadowing represents the standard deviations obtained from all measurements performed over time.

The translational and rotational corrections of source and detector obtained during the weekly calibrations are shown in Figure 5. The results are provided for the clockwise gantry rotation direction. The counter‐clockwise direction revealed similar results that are not reported for brevity. Particularly the detector corrections showed reasonable stability over time, with maximum angle‐specific standard deviations of 1.7 mm (obtained for tC*_x_*) and 0.11° (r*_y_*). Averaged over all angles, the standard deviations amounted at maximum 1.3 mm (tC*_x_*) and 0.08° (r*_y_*). The translational corrections of the X‐ray source revealed higher variations, with a maximum and maximum mean (averaged over all angles) standard deviation of 3.4 mm (tS*_x_*) and 2.3 mm (tS*_x_*), respectively.

### 3.4 Laser/jaw accuracy

**FIGURE 6:**
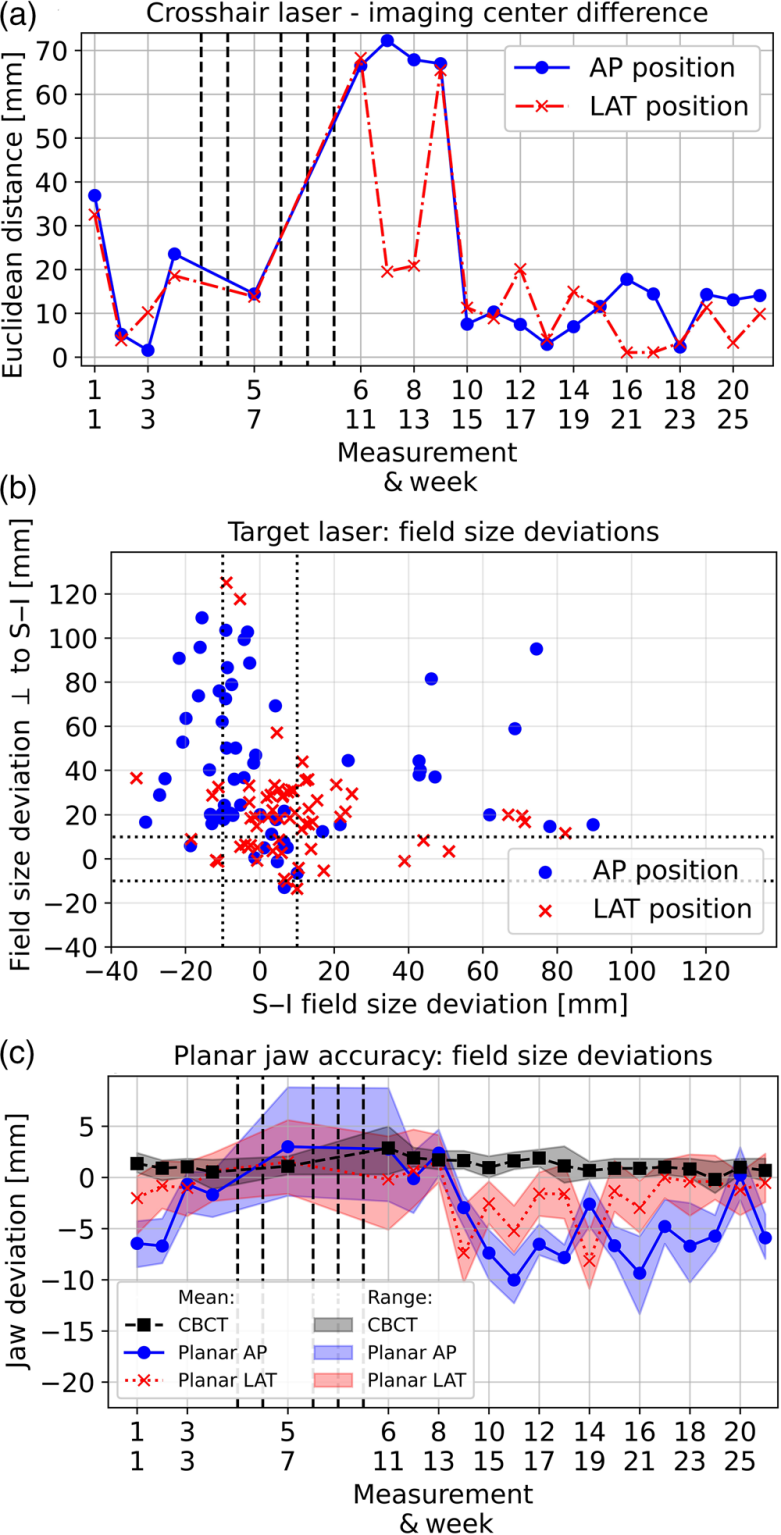
Laser and jaw stability: Part (a) shows the Euclidean distances between crosshair‐laser and imaging center for both imaging positions examined over time. In (b), the deviations of measured to set field sizes are shown conclusively for all weeks and all field sizes examined. Results are presented separately for both the anterior–posterior (AP)‐ and lateral (LAT)‐imaging positions. The dotted lines indicate our desired tolerance of ±10 mm. Jaw accuracy (c) is shown for both planar and cone‐beam computed tomography (CBCT) imaging, with the shadowing providing the range of the results. For planar‐imaging, six measurements (three field size deviations in superior–inferior (S–I) direction and orthogonal direction, respectively) were considered in each week for both imaging positions. In (a) and (c), the dashed lines indicate the weeks in which no measurements could be performed.

Laser accuracy and precision were very low. The crosshair‐laser deviated non‐reproducible several centimeters from the imaging center (Figure 6a). Between the fifth (after maintenance) and ninth week (laser replacement), even more than 70 mm deviations occurred. The target‐laser also showed substantial differences between projected and set fields, which are shown conclusive in Figure 6b for all measurements and set field sizes. Deviations in superior–inferior direction and the direction orthogonal to it (LAT direction for AP imaging position; AP direction for LAT position) were analyzed separately. We observed field size variations of up to more than 120 mm, which were more pronounced for the AP than for the LAT imaging position and clearly exceeded our desired tolerance of 10 mm (Figure 6b; dotted lines). Thus, both crosshair‐ and target‐laser showed a dependency on imaging position (AP/LAT) and were generally non‐reproducible.

The analysis of jaw accuracy revealed substantial differences between planar and CBCT‐imaging. For CBCT‐imaging, a maximum deviation of measured and set jaw positions of 5 mm was obtained (Figure 6c). Averaged over time, the jaw deviations amounted 1.2 ± 0.7 mm. However, the maximum jaw position deviation in planar‐imaging was −13 mm. The mean deviation over time amounted −4.0 ± 4.0 mm and −1.6 ± 2.6 mm for the AP‐ and LAT‐imaging position, respectively. The imaged field sizes were thus generally smaller than the ones set and jaw accuracy depended on the IRm's imaging position (Figure 6c). No general difference between the laser deviations along both field size directions was found.

## 4 DISCUSSION

In this work, we presented our QA procedure for a novel 3‐in‐1 X‐ray system and provided the first assessment regarding its long‐term stability. The results serve as baseline/comparison for the ongoing IRm QA and help to detect performance alterations over time early. This is crucial to ensure performance stability in clinical operations. Our workflow may also serve as a starting point for the QA of future 3‐in‐1 systems, as, to our knowledge, currently no comparable devices with such innovative technologies as movable lasers and dynamic jaws exist. The IRm sets new technological standards in this respect.

Regarding planar‐ and CBCT‐imaging, our results showed reasonable stability over time and good agreement with phantom specifications.^24^ For CBCT‐imaging, the latter is in accordance with a previous publication.^12^ Image uniformity depended on the used scan protocol, as the IRm requires an explicit manual adaption of heuristic scatter parameters to imaging parameters as pre‐filtration or tube voltage for each single protocol. For planar imaging, five detail objects and (after week 5) all staircase steps were detectable in each case. This exceeds the DIN guidelines^16^ with 4‐5 objects and 14–16 steps recommended to be detectable. Thus, the IRm provided good image quality in general. This holds particularly for the time after the maintenance (week 5), where improvements in post‐processing for planar‐imaging and scatter corrections for stitched full scans resulted in the reported image quality improvements. However, as drawback, the weekly varying CBCT‐artifacts have to be noted as well. The streaks were assumed to originate from tube output and/or detector read‐out instabilities but still have to be investigated further.

The varying misalignment artifacts could be an indicator for mechanical device instabilities. The obtained correction variations of up to 3.4 mm (tS*_x_*) were, due to a CBCT sampling distance of 180 μm in the gantry center, not negligible. Geometric uncertainties of the IRm assumed to originate from mechanical instabilities have also been reported by Karius et al.^12^ Nevertheless, our results are improved against this previous study reporting variations of up to 5.3 mm (tS*_z_*) and 0.61° (r*_y_*). We conclude that substantial manufacturer‐sided improvements regarding the geometric stability must have been carried out in the meantime. These will have to be extended to increase stability further.

The IRm lasers worked non‐reproducible and were, due to the large deviations from set positions, not suitable for the reliable patient alignment within the scanner. For this purpose and reason, we currently use additional external lasers in our institution. Furthermore, a tolerable jaw accuracy of 5 mm was achieved for CBCT‐imaging, whereas much larger position deviations were obtained for planar imaging. This is of particular clinical relevance, as the obtained results will have to be considered as safety margins in adjusting the FOV of examinations to ensure that the entire anatomical ROI is captured sufficiently on the scans. Note that the maximum jaw inaccuracies were in each case worse than suggested by the AAPM TG‐142 report^25^ for symmetric jaw positioning in linear accelerators, which allows the positioning tolerances of 2 mm. The reasons for the deviations in jaw positioning between planar‐ and CBCT‐imaging could not be identified yet and require, as for imperative laser improvements, further consultations with and solutions by the manufacturer.

Based on the reasonable image quality stability, we continue to perform the described QA for planar‐ and CBCT‐imaging and geometric stability once a month. We consider the image quality parameters presented as appropriate for a profound assessment of the system stability over time. The IRm was operated clinically during the time of measurements for this work without any major imaging drawbacks, and thus the clinical impact of the physical image quality variations was considered tolerable. Based on our results and clinical experience, we defined in our institution current tolerance levels for CT‐number accuracy, CNR, uniformity, spatial resolution, geometric accuracy, and CBDI_w_ of ±50 HU, ±50%, ±50 HU, ±1 lp/cm, ±1 mm, and ±1 mGy (with respect to the baselines) in CBCT‐imaging. For planar‐imaging, we chose tolerances of ±1 μGy, ±0.2 lp/mm, at minimum four detectable objects, and at minimum 14 identified steps for dose, spatial resolution, detectability, and dynamic range, respectively. The latter two values are particularly also recommendations of the current DIN guidelines^16^ for planar‐imaging assessment. For geometric stability, it is desired that the acquired curves for each of the nine flexmap correction parameters do not deviate by more than three times the standard deviations from the mean curves obtained in this work. The QA of jaw‐positioning accuracy will be continued on a weekly basis, due to the higher fluctuations of the corresponding results and to detect severe performance deteriorations early. Here, we use respective tolerances for jaw‐positioning deviations from the set field sizes of ±5 mm and ±10 mm for CBCT‐ and planar‐imaging, respectively. Regarding the lasers, the reported non‐reproducibility makes a QA pointless at present and significant improvements are required first, but maximum field size deviations of <10 mm would be desirable. For completion, it has to be noted that even exceeding individual tolerances may be acceptable and that a holistic view of all investigated (imaging) performance parameters by experienced physicists or technicians is required to evaluate the final outcome of the QA procedure. This holds particularly true for the decision to take additional measures (e.g., scanner re‐calibration). Continuous documentation of the results is considered crucial to detect even creeping performance alterations over time early.

## 5 CONCLUSION

We developed a QA workflow for a novel 3‐in‐1 X‐ray system and assessed its planar and CBCT‐imaging performance regarding image quality and dose, geometric stability, dynamic jaws, and motorized lasers. Although the imaging performance and geometric stability showed a reasonable stability over time, laser, and jaw‐positioning accuracy need significant improvements.

## CONFLICT OF INTEREST

The authors have no financial or non‐financial interests to disclose.

## AUTHOR CONTRIBUTIONS

All authors contributed significantly to the performed work and approved the manuscript to be published.

## Supporting information

Supplementary Materials A

Supplementary Material B

## Data Availability

The data that support the findings of this study are available from the corresponding author upon reasonable request. The work was not published previously and is not submitted elsewhere.
