# Supplementary material for: Quality assurance and long‐term stability of a novel 3‐in‐1 X‐ray system for brachytherapy
Source: J Appl Clin Med Phys. 2022 Jul 18;23(9):e13727. doi: 10.1002/acm2.13727 (PMC9512339; doi:10.1002/acm2.13727)
Supplement: Supplementary file 1 — Supplementary Materials A [file ACM2-23-e13727-s001.docx]

# Supplementary Materials A

*Shown are the baselines obtained during the* ***very first measurement*** *for each CBCT-imaging parameter examined.*

| **Insert** | **Pelvis protocol** | **Breast protocol** | **Abdomen protocol** |
| --- | --- | --- | --- |
|  | **CT-numbers [HU]** | | |
| - Air | -957 | -784 | -983 |
| - PMP | -187 | -186 | -234 |
| - LDPE | -101 | -119 | -168 |
| - Polystyrene | -41 | -76 | -119 |
| - Acrylic | 110 | 35 | 36 |
| - Delrin® | 330 | 198 | 247 |
| - Teflon® | 927 | 605 | 764 |
|  | **Contrast-noise-ratio []** | | |
| - LDPE | 15 | 7 | 12 |
| - Polystyrene | 12 | 11 | 14 |
| - Acrylic | 3 | 1 | 1 |
|  | **Geometric accuracy [mm]** | | |
|  | -0.17 ± 0.20 | -0.23 ± 0.10 | -0.49 ± 0.16 |
|  | **Limiting spatial resolution [lp/cm]** | | |
|  | 12 | 11 | 8 |
|  |  | **Uniformity [HU]** |  |
|  | -23 | 127 | 223 |
|  | **Weighted cone-beam dose index CBDI_w_ [mGy]** | | |
|  | 11.3 | 5.8 | 9.4 |

*Shown are the baselines obtained during the* ***very first measurement*** *for each planar imaging parameter examined.*

| **Planar imaging parameter** | **Pelvis protocol** | **Breast protocol** | **Abdomen protocol** | **Fluoroscopy** |
| --- | --- | --- | --- | --- |
| Limiting resolution [lp/mm] | 1.0 | 1.2 | 1.2 | 1.8 |
| Number of  detected steps | 16 | 11 | 15 | 14 |
| Number of detected detail objects | 5 | 5 | 5 | 5 |
| Detector entrance dose [µGy] | 2.74 | 1.58 | 3.26 | 278.4 |

*Shown are the results obtained* ***after maintenance*** *for each CBCT-imaging parameter examined.*

| **Insert** | **Pelvis protocol** | **Breast protocol** | **Abdomen protocol** |
| --- | --- | --- | --- |
|  | **CT-numbers [HU]** | | |
| - Air | -986 | -800 | -965 |
| - PMP | -202 | -189 | -199 |
| - LDPE | -106 | -113 | -108 |
| - Polystyrene | -38 | -72 | -77 |
| - Acrylic | 115 | 42 | 75 |
| - Delrin® | 354 | 203 | 305 |
| - Teflon® | 986 | 624 | 849 |
|  | **Contrast-noise-ratio []** | | |
| - LDPE | 14 | 7 | 15 |
| - Polystyrene | 12 | 7 | 13 |
| - Acrylic | 2 | 0 | 2 |
|  | **Limiting spatial resolution [lp/cm]** | | |
|  | 12 | 11 | 8 |
|  |  | **Uniformity [HU]** |  |
|  | -18 | 129 | 177 |
|  | **Weighted cone-beam dose index CBDI_w_ [mGy]** | | |
|  | 12.0 | 5.7 | 9.5 |

*Shown are the results obtained* ***after maintenance*** *for each planar imaging parameter examined.*

| **Planar imaging parameter** | **Pelvis protocol** | **Breast protocol** | **Abdomen protocol** | **Fluoroscopy** |
| --- | --- | --- | --- | --- |
| Limiting resolution [lp/mm] | 1.0 | 1.2 | 1.2 | 1.8 |
| Number of  detected steps | 17 | 17 | 17 | 17 |
| Number of detected detail objects | 5 | 5 | 5 | 5 |
| Detector entrance dose [µGy] | 2.58 | 1.46 | 2.8 | 258.8 |
