# Supplementary material for: Quality assurance and long‐term stability of a novel 3‐in‐1 X‐ray system for brachytherapy
Source: J Appl Clin Med Phys. 2022 Jul 18;23(9):e13727. doi: 10.1002/acm2.13727 (PMC9512339; doi:10.1002/acm2.13727)

# Supplementary Material B

*As reported in the manuscript, severe and variable misalignment and streak artifacts occurred in CBCT-imaging. For illustration, we provide in the following 10 scans of the CatPhan’s CTP404 module acquired exemplarily with the pelvis CBCT-protocol. The obtained results are shown in steps of two measurements here. Window Level: 40 HU, Width: 600 HU.*


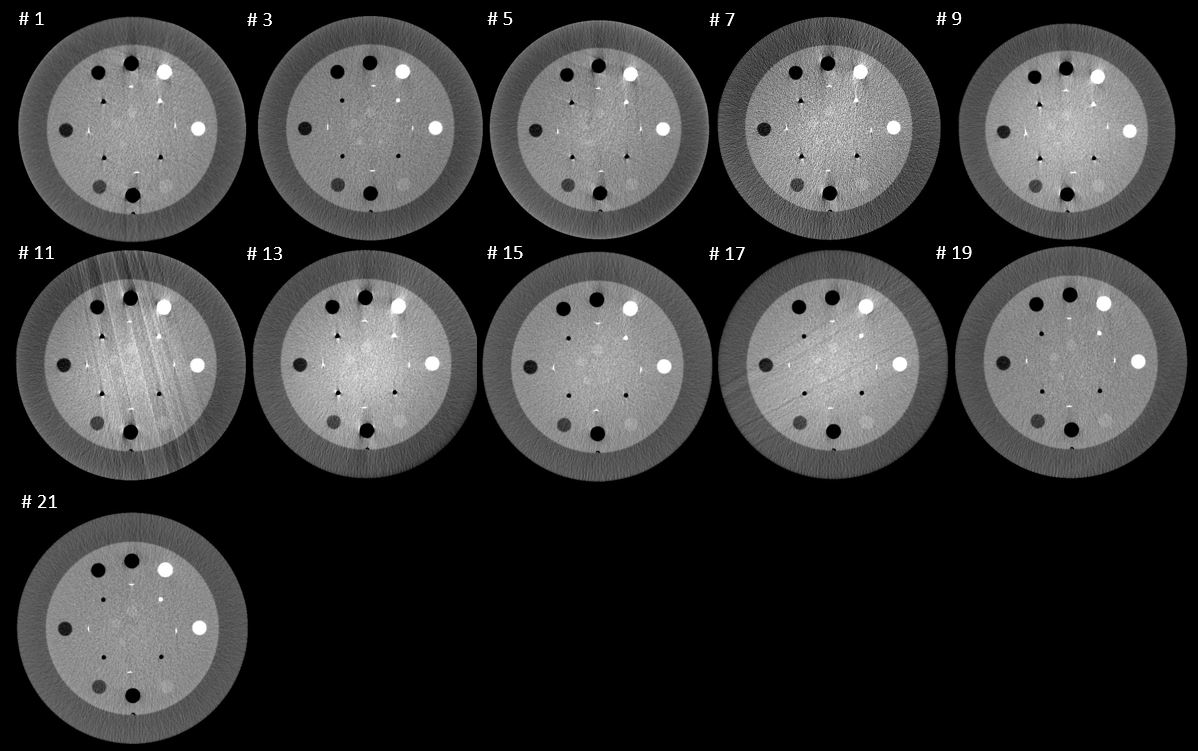

Supplement: Supplementary file 2 — Supplementary Material B [file ACM2-23-e13727-s002.docx]
